# Transcriptional profiling of wheat (*Triticum aestivum* L.) during a compatible interaction with the cereal cyst nematode *Heterodera avenae*

**DOI:** 10.1038/s41598-018-37824-9

**Published:** 2019-02-18

**Authors:** Fen Qiao, Ling-An Kong, Huan Peng, Wen-Kun Huang, Du-Qing Wu, Shi-Ming Liu, Jihong Liu Clarke, De-Wen Qiu, De-Liang Peng

**Affiliations:** 1 State Key Laboratory for Biology of Plant Diseases and Insect Pests, Institute of Plant Protection, Chinese Academy of Agricultural Sciences, Beijing, 100193 China; 2 NIBIO - Norwegian Institute of Bioeconomy Research, P.O. Box 115, N-1431 Oslo, Ås Norway

## Abstract

Cereal cyst nematode (CCN, *Heterodera avenae*) presents severe challenges to wheat (*Triticum aestivum* L.) production worldwide. An investigation of the interaction between wheat and CCN can greatly improve our understanding of how nematodes alter wheat root metabolic pathways for their development and could contribute to new control strategies against CCN. In this study, we conducted transcriptome analyses of wheat cv. Wen 19 (Wen19) by using RNA-Seq during the compatible interaction with CCN at 1, 3 and 8 days past inoculation (dpi). In total, 71,569 transcripts were identified, and 10,929 of them were examined as differentially expressed genes (DEGs) in response to CCN infection. Based on the functional annotation and orthologous findings, the protein phosphorylation, oxidation-reduction process, regulation of transcription, metabolic process, transport, and response process as well as many other pathways previously reported were enriched at the transcriptional level. Plant cell wall hydrolysis and modifying proteins, auxin biosynthesis, signalling and transporter genes were up-regulated by CCN infection to facilitate penetration, migration and syncytium establishment. Genes responding to wounding and jasmonic acid stimuli were enriched at 1 dpi. We found 16 *NBS-LRR* genes, 12 of which were down-regulated, indicating the repression of resistance. The expression of genes encoding antioxidant enzymes, glutathione S-transferases and UDP-glucosyltransferase was significantly up-regulated during CCN infection, indicating that they may play key roles in the compatible interaction of wheat with CCN. Taken together, the results obtained from the transcriptome analyses indicate that the genes involved in oxidation-reduction processes, induction and suppression of resistance, metabolism, transport and syncytium establishment may be involved in the compatible interaction of Wen 19 with CCN. This study provides new insights into the responses of wheat to CCN infection. These insights could facilitate the elucidation of the potential mechanisms of wheat responses to CCN.

## Introduction

Wheat (*Triticum aestivum* L.) is one of the most widely grown staple crops, providing a major source of energy and dietary fiber for humans^1^. Cereal cyst nematode (CCN, *Heterodera avenae*) is one of the most devastating sedentary endoparasitic pests and causes large yield declines in wheat production worldwide^2^. In China, the CCN occurs in 80% of the wheat growing areas and causes annual yield losses of 20–30%^3^. During the CCN life cycle, second-stage juveniles (J2) hatch from eggs at an optimum temperature of 16 °C after a low temperature (approximately 4 °C) treatment for at least 40 days. The J2 larvae invade from an elongation zone and migrate intracellularly in the wheat root with the help of their secreted cell wall softening enzymes^4^. The J2 larvae then select a cell adjacent to the host vascular tissues and establish a nematode feeding site (NFS) approximately 3 to 4 days past infection^5,6^. Subsequently, the NFS develops and lesds to the formation of a syncytium, which serves as the sole nutrient source for the sedentary life stages^7^. The J2s undergo three moults to develop into male or female adults, prior to fertilization and completion of their reproductive cycle^2,8^.

These nematodes have evolved a special capacity to secrete effectors and dramatically manipulate the host functions for their own development^9^. Compatible plant-nematode interactions have revealed a large number of genes and pathways associated with nematode infection, which greatly helps us understand the parasitic mechanism of the nematode. Several high-throughput methods have been employed to analyse the transcriptional regulation of the host genes by nematode parasitism including microarrays^10–12^, proteomic analysis^13^ and RNA-Seq^6,14^. Microarray analysis has been used to study the compatible interactions of soybean cyst nematode (SCN, *H. glycines*) with soybean (*Glycine max* L.). The results revealed that the genes encoding trehalose phosphate synthase and phospholipase D and the genes involved in metabolism, transport and resistance were induced in susceptible soybean plants^12^. The compatible interaction of *Musa acuminata* and root-knot nematode (RKN, *Meloidogyne incognita*) led to early host defence responses including reactive oxygen species and jasmonate/ethylene plant hormone signalling^15^. Auxin metabolism and cell wall modification genes were also induced^15^. Host transcriptional profiling of *Phaseolus vulgaris* in response to RKN during compatible interactions showed that 797 host genes were differentially expressed; the resistance was repressed, and reactive oxygen species were reduced^16^. The transcriptome response of rice to sedentary endoparasitic RKN and migratory root rot nematode (RRN, *Hirschmanniella oryzae*) showed that RKN suppress the salicylic acid and ethylene pathways, stimulate host metabolism, and strongly induce the expression of plant development-associated hormones, while the migratory nematode (RRN) induces program cell death and oxidative stress^17^. In addition, the previous studies conducted on fungal diseases, e.g., compatible interactions between wheat and *Septoria tritici*^18^ and between tomato and *Phytophthora infestans*^19^, broadened our knowledge of the infection process. Analyses of resistant and susceptible host genotypes in response to CCN infection has shown that the resistance does not disturb the penetration stages of CCN but does affect it through the developmental stages; the phospholipases may play a role in defence responses, and there is a strong burst of reactive oxygen species in resistant wheat after infection by CCN^6^. Comparative transcriptome analyses of soybean-SCN interactions has shown that ethylene, protein degradation, and phenylpropanoid pathways are important for resistance^20^. An investigation on the interaction of wild soybean with SCN interactions has shown that plant hormone signalling, MAPK signalling and defence signalling were affected in both resistant and susceptible genotypes^21^.

Little is known about compatible wheat responses to CCN parasitism during invasion, the establishment and maintenance of feeding sites. Information about CCN-responsive genes and pathways is lacking. In this study, we applied RNA-Seq technology to investigate the compatible interaction of wheat and CCN at three early time points. This approach was used not only to characterize the differentiallly expressed genes (DEGs) during CCN penetration but also to examine the gene response to feeding site establishment and maintenance. Our study provides novel data on comprehensive gene expression profiling of the compatible wheat and CCN interaction, which will be important to reveal the underlying infection mechanism and to effectively manage CCN in the future.

## Results

### Identification and functional annotation of differentially expressed genes

In our previous studies, to identify CCN resistance candidate genes, the transcriptome of Wen 19 in response to CCN was sequenced by RNA-Seq^6^. To generate comprehensive information during the compatible interaction between *T. aestivum* L. and *H. avenae*, this dataset was used to explore compatible transcriptional analysis of Wen 19 in response to the CCN at three time points: 1 dpi, 3 dpi and 8 dpi. Similar numbers of clean reads were obtained at each time point: 214,092,190, 215,995,184 and 215,455,900, respectively (Table S1). The DEGs between the control (before nematode infection) and nematode-infected roots were defined as two-fold up or down-regulated genes with FDR correction and a *P* value ≤ 0.001. In this manner, we found the largest numbers of DEGs (6,029) at the time point of feeding site establishment (3 dpi), including 4,467 up-regulated and 1,563 down-regulated genes, followed by the penetration stage (1 dpi), at which 3,649 DEGs were detected including 2,513 up-regulated and 1,136 down-regulated genes. At the development stage of the feeding site (8 dpi), only 1,251 significant DEGs were found, including 945 up-regulated and 306 down-regulated genes (Table S1), suggesting that the Wen 19 may have a strong reaction during the early infection stages of CCN (1 dpi and 3 dpi) and that the CCN was able to inhibit the wheat responses in the compatible interaction at 8 dpi (Table S1). To search the primary functional groups of the DEGs, we performed BLASTx alignments to protein databases Nr, Swiss-Prot, KEGG and COG with a cut-off E-value of 1e-5. This indicates that a total of 10,929 (81.72%), 9,247 (79.55%), 7,270 (51.06%) and 5,437 (49.75%) DEGs had significant matches in the Nr, Swiss-Prot, KEGG and COG databases, respectively (Table 1).

**Table 1:** Functional annotation of the differentially expressed genes (DEGs) of Wen 19 in response to the CCN.

CCN response	No. of DEGs	No. of DEGs in NR	No. of DEGs in Swiss-Prot	No. of DEGs in KEGG	No. of DEGs in COG
1 dpi	3649	2982	2377	1136	1507
3 dpi	6029	5228	4103	3747	2553
8 dpi	1251	1037	790	697	477
Total	10929	9247 (81.72%)	7270 (79.55%)	5580 (51.06%)	5437 (49.75%)

**Figure 1:**
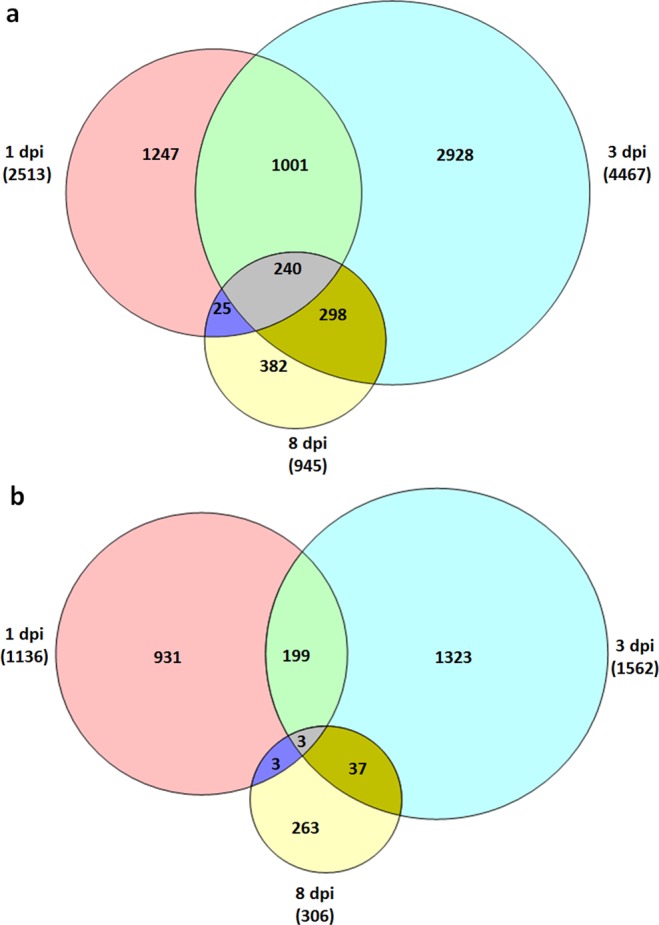
Venn diagram of the numbers of up-regulated (**a**) and down-regulated (**b**) genes at 1 dpi, 3 dpi and 8 dpi.

Among the 7,925 up-regulated genes, 240 genes were up-regulated at all three time points, 1,564 genes at two time points (1,241 genes at 1 and 3 dpi, 265 at 1 and 8 dpi, and 538 at 3 and 8 dpi) and 4,557 at one time point (1247, 2928 and 382 at 1 dpi, 3 dpi and 8 dpi, respectively). The numbers of exclusive genes belonging to were (Fig. 1a). Three genes were significantly down-regulated at all three time points (Fig. 1b), while 242 genes were down-regulated at two time points (202 genes at 1 and 3 dpi, 6 genes at 1 and 8 dpi and 40 genes at 3 and 8 dpi) and 2570 genes were down-regulated at one time point (931, 1,323 and 263 at 1 dpi, 3 dpi and 8 dpi, respectively) (Fig. 1b).

### Transcriptome changes in the root tissue at 1 dpi

**Figure 2:**
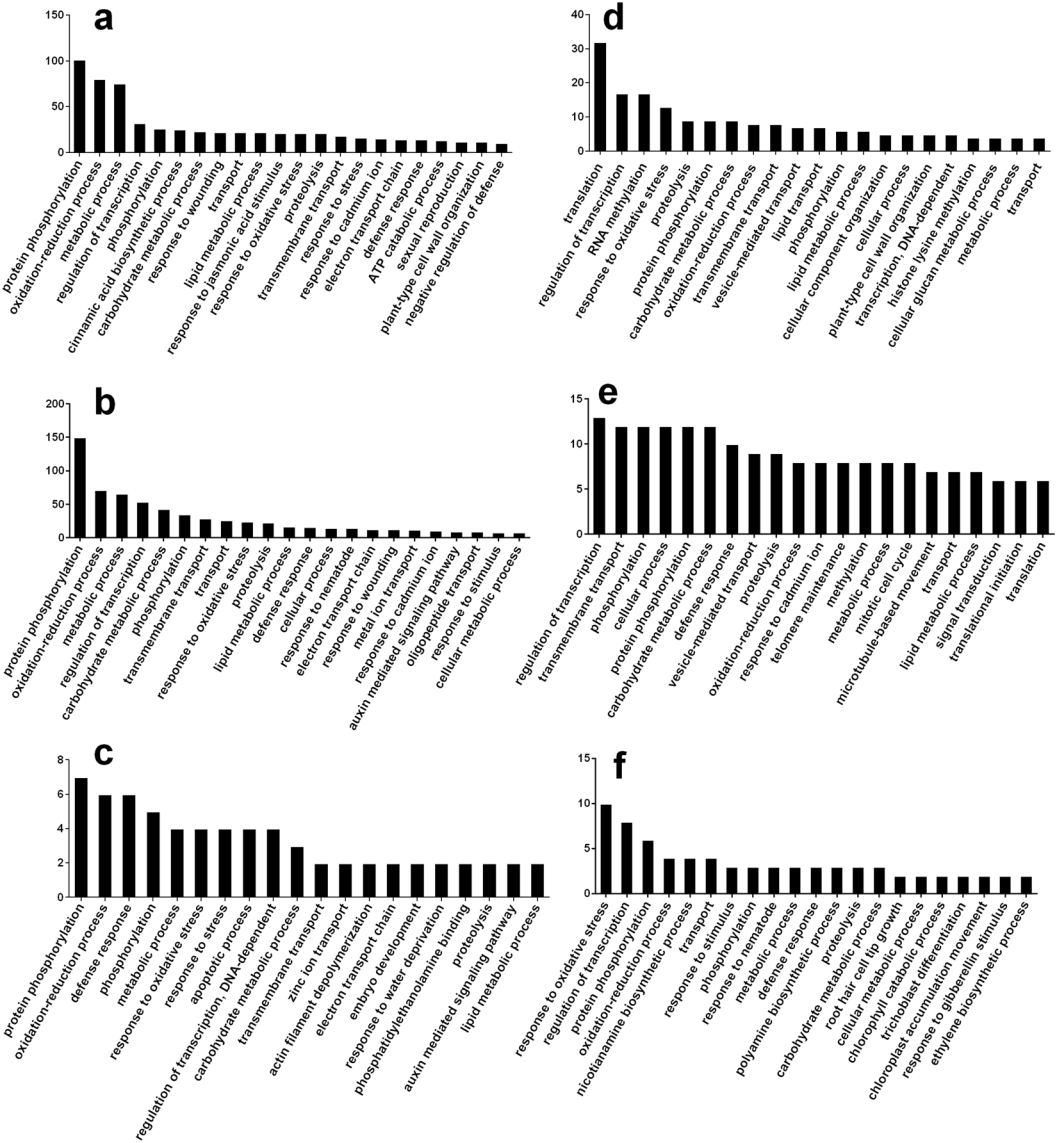
Gene ontology (GO) biological process of Wen 19 during the compatible interaction with the CCN at 1 dpi (**a,d**), 3 dpi (**c,e**) and 8 dpi (**c,f**). Numbers of up-regulated (**a**–**c**) and down-regulated (**d**–**f**) genes are illustrated in the y-axis.

At 1 dpi when the CCN had penetrated the root, 2,178 DEGs (FDR <0.001) were exclusively found. Of these, 1,247 genes were up-regulated and 931 genes were down-regulated (Fig. 1; Table S1). Gene enrichment analysis showed 101 genes involved in protein phosphorylation, 75 genes involved in metabolic processes, 40 genes involved in transporter and transmembrane transport, and 32 genes involved in the regulation of transcription were up-regulated at 1 dpi (Fig. 2a). Plant cell wall modification may facilitate penetration and movement. We found that 12 genes in plant type cell wall organization were induced by the CCN (Fig. 2a). Genes involved in the oxidation-reduction process, in response to wounding and jasmonic acid stimuli were greatly up-regulated, which may reflect the induced host resistance at the early stage of the CCN invasion (Fig. 2a). The strongest up-regulated genes were the genes encoding aromatic-L-amino-acid decarboxylase, beta-sesquiphellandrene synthase, UDP-glycosyltransferase 91B1, nodulation-signalling pathway 2 protein, and probably linoleate 9S-lipoxygenase 4 (Table S2). The genes for the GO terms of translation, regulation of transcription, RNA methylation, response to oxidative stress, proteolysis, vesicle-mediated transport, and lipid metabolic processes were down-regulated in the infected roots (Fig. 2d). The genes that were strongly down-regulated were two genes encoding a dehydration-responsive element-binding protein and a senescence-induced receptor-like serine/threonine-protein kinase (Table S2).

### Transcriptome changes in the root tissue at 3 dpi

A total of 4,251 DEGs (FDR <0.001) were found exclusively at 3 dpi when the feeding sites were established. Of these, 2,928 genes were up-regulated, and 1,323 genes were down-regulated (Fig. 1, Table S1). GO terms enrichment analysis showed that genes involved in protein phosphorylation, the oxidation-reduction process, regulation of transcription, metabolic processes, and transmembrane transport were significantly enriched in up- and down-regulated genes at 3 dpi (Fig. 2b,d). Auxin is a key element for syncytium formation^22^. In our dataset, the genes in the auxin-mediated signalling pathway were up-regulated (Fig. 2b). The genes encoding glutathione S-transferase GSTF1, bidirectional sugar transporter SWEET12, ethylene-responsive transcription factor RAP2-2, indole-2-monooxygenase, and peptide transporter PTR2 were strongly up-regulated (Table S2), while those involved in vesicle-mediated transport, signal transduction and translation were down-regulated in the infected root (Fig. 2d). The genes encoding ethylene-responsive transcription factor TINY, transcription factor HY5, and phytochrome-associated serine/threonine-protein phosphatase were strongly down-regulated (Table S2).

### Transcriptome changes in root tissue at 8 dpi

In total, 645 DEGs (FDR <0.001) were exclusively found at 8 dpi. Of these, 382 genes were up-regulated and 263 genes were down-regulated (Fig. 1, Table S1). GO enrichment revealed that the genes involved in protein phosphorylation, response to stress, the oxidation-reduction process, defence response and metabolic process, transport, and response to stimulus were significantly affected upon CCN infection (Fig. 2c,f), and the genes involved in the regulation of transcription and the nicotiana mine biosynthetic process were down-regulated at 8 dpi (Fig. 2f). Alternatively, genes involved in zinc ion transport and the auxin mediated signalling pathway were up-regulated at 8 dpi (Fig. 2c). The strongest up-regulated genes were those encoding proline-rich receptor-like protein kinase PERK10, ethylene responsive transcription factor 6, and cytosolic sulfotransferase 12. The strongest down-regulated genes were those encoding beta-galactosidase 7, elongation factor 1-alpha C and uncharacterized proteins (Table S2).

### Plant cell wall, plant hormone auxin and jasmonic acid play important roles in CCN infection

Both root penetration and syncytium establishment depend on the degradation and modification of the host cell wall^23,24^. In our dataset, the genes involved in plant cell wall hydrolysis and modifying proteins such as xyloglucan endotransglycosylase/hydrolase protein 32, expansins, endoglucanase 2 and endoglucanase 4 were up-regulated by CCN infection at 1 dpi and 3 dpi (Table 2). The genes encoding expansin-B7, expansin-A3 and probable pectate lyase 8 were up-regulated by CCN infection at 1 dpi, 3 dpi and 8 dpi (Table 2). The genes encoding xyloglucan endotransglycosylase/hydrolase protein 8 and pectate lyase were up-regulated at 3 dpi, while the genes encoding probable xyloglucan endotransglucosylase/hydrolase and expansin-A9 were down-regulated at 1 dpi and 3 dpi (Table 2). In jasmonic acid (JA) biosynthesis and signalling, the probable linoleate 9S-lipoxygenase 4 and linoleate 9S-lipoxygenase 1 genes were intensely induced at 1 dpi but not in the later infection stages. The genes encoding allene oxide cyclase 3 and pathogenesis-related protein PRB1–2 were only induced at 3 dpi (Table 3). The genes encoding a lipoxygenase 6 and a linoleate 9S-lipoxygenase 2 were up-regulated at 1 and 3 dpi, while another linoleate 9S-lipoxygenase 2 gene was down-regulated at 3 and 8 dpi. Lipoxygenase 8 and probable linoleate 9S-lipoxygenase 5 genes were up-regulated at 1 dpi, 3 dpi and 8 dpi (Table 3). The gene set of the auxin-mediated signalling pathway was up-regulated at 3 dpi and 8 dpi (Fig. 2b,c). Genes involved in auxin biosynthesis, such as those encoding flavin-containing monooxygenase YUCCA9, indole-3-acetic acid-amido synthetase, aromatic-L-amino-acid decarboxylase and auxin response and induced proteins were up-regulated by CCN at 1 dpi and 3 dpi (Table 3). A gene encoding an auxin influx carrier was significantly up-regulated, but one encoding the auxin efflux carrier component 1c was down-regulated (Table 3).

**Table 2:** Expression of genes involved in cell wall hydrolysis and encoding modifying proteins in the infected Wen19 compared to the non-infected Wen 19. FC: fold change (CCN infection *vs* non-infected samples);–: not differentially expressed.

GenBand-ID	Annotation	Log_2_FC 1 dpi	Log_2_FC 3 dpi	Log_2_FC 8 dpi
MH883021	Expansin-B2	1.4162	2.4476	1.0598
MH908106	Expansin-B4	2.738	3.8566	—
MH908107	Expansin-B3	2.326	1.8641	—
MH908108	Expansin-B2	2.6025	3.5502	—
MH908109	Expansin-B15	1.2109	1.415	—
MH908110	Expansin-A8	2.8025	2.2812	—
MH908111	Expansin-A7	1.7415	1.9023	—
MH908112	Expansin-A3	3.0243	3.7292	2.1655
MH908113	Expansin-A2	1.717	2.246	—
MH908114	Expansin-A1	1.7882	2.2398	—
MH908115	Xyloglucan endotransglycosylase/hydrolase protein 8	—	1.2156	—
MH908116	Probable xyloglucan endotransglucosylase/hydrolase protein 32	2.3702	1.4671	—
MH908117	Pectate lyase	—	1.0949	—
MH908118	Endonuclease 4	1.3031	1.3742	—
MH908119	Endonuclease 2	1.7503	1.2929	—
MH908120	Probable xyloglucan endotransglucosylase/hydrolase	−2.6012	−1.7502	—
MH908121	Expansin-A9	−1.3792	−1.3956	—

**Table 3:** Expression of the genes involved in jasmonic acid and auxin signalling pathways in the infected Wen19 compared to the non-infected Wen 19. FC: fold change (CCN infection *vs* non-infected samples);–: not differentially expressed.

GenBank-ID	Annotation	Log_2_FC 1 dpi	Log_2_FC 3 dpi	Log_2_FC 8 dpi
**Jasmonic acid signalling pathway**				
MH891149	Probable lipoxygenase 8, chloroplastic	2.6551	3.7291	1.7409
MH891150	Probable linoleate 9S-lipoxygenase 5	4.3855	5.9062	1.3881
MH891151	Probable lipoxygenase 6	1.4287	1.3063	—
MH891153	Allene oxide cyclase 3, chloroplastic	1.3774	1.6346	—
MH891152	Pathogenesis-related protein PR1–2	—	1.9079	—
MH891154	Linoleate 9S-lipoxygenase 1	2.1548	—	—
**Auxin signalling pathway**				
MH891155	Aromatic-L-amino-acid decarboxylase	6.0844	4.9628	2.3992
MH891156	Flavin-containing monooxygenase FMO	—	1.7399	—
MH891157	Flavin-containing monooxygenase YUCCA9	1.2702	2.4058	—
MH891158	Indole-3-acetic acid-induced protein ARG7	—	1.4409	—
MH891159	Auxin-induced in root cultures protein 12	2.4763	3.6903	—
MH891160	Auxin response factor 5	—	1.3689	—
MH891161	Auxin-repressed 12.5 kDa protein	—	1.4916	—
MH891162	Auxin-responsive protein IAA21	—	1.1678	—
MH891163	Auxin-induced protein X15	—	1.474	—
MH891164	Auxin-induced protein 10A5	1.2925	1.4842	—
MH891165	WAT1-related protein	2.2927	2.7679	1.688
MH891166	Auxin influx carrier (AUX1 LAX family)	—	2.015	1.6046
MH891167	Probable auxin efflux carrier component 1c	—	−1.6004	—

### CCN-responsive genes in antioxidant and detoxification processes

**Figure 3:**
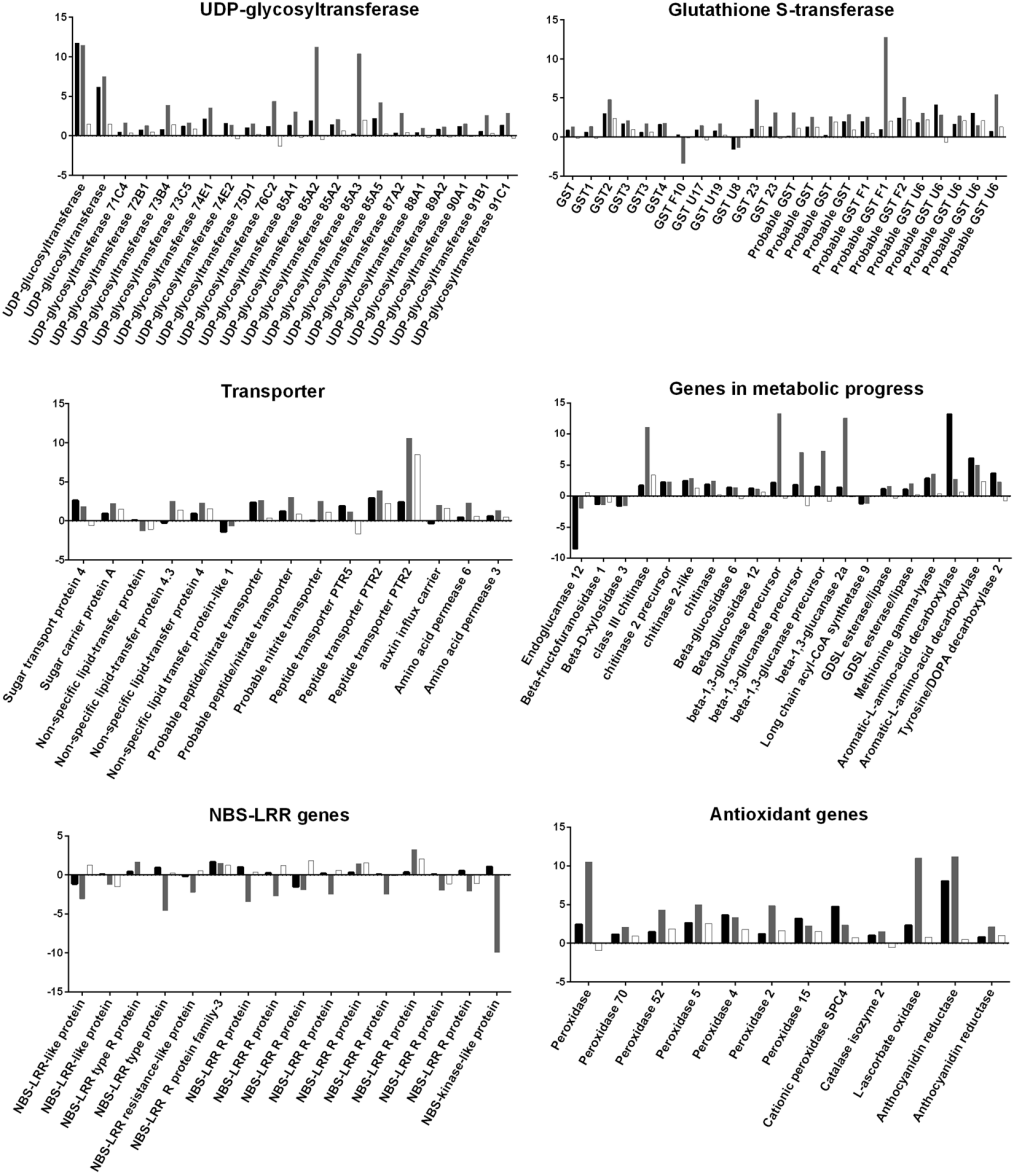
Expression profiles of UDP-glucosyltransferase, GST (glutathione S-transferase), transporter, metabolic progress, and *NBS-LRR* genes from the RNA-Seq data. Units are normalized to the numbers of reads. Log_2_FC between nematode-infected and non-infected (control) samples at each time point is indicated in the y-axis. FC: fold change (CCN infection *vs* control samples).  : 1 dpi  : 3 dpi  : 8 dpi.

The GO biological process enrichment showed that genes involved in the oxidation-reduction process and in response to oxidative stress were enriched in both up- and down-regulated genes at all three time points tested. After pathogen recognition, reactive oxygen species (ROS) are rapidly induced and accumulated^6^. In this study we found that eight respiratory burst oxidase genes were induced by CCN infection and were especially highly expressed at 3 dpi (Table S3). Genes encoding peroxidase and the antioxidant enzymes ascorbate and anthocyanidin reductase were induced (Fig. 3; Table S3). Detoxification genes, such as those encoding UDP-glycosyltransferase and glutathione S-transferases (GST) were highly expressed at the early stages. We found 24 DEGs in the RNA-Seq data which were annotated to encode glutathione S-transferases in the Nr or Swissprot database. Of these, 12, 22 and 11 were significantly up-regulated at 1 dpi, 3 dpi and 8dpi respectively (Fig. 3; Table S4). Among the differentially expressed UDP-glycosyltransferase genes, 11 and 14 were significantly up-regulated at 1 dpi and 3 dpi, respectively (Fig. 3; Table S4). In addition, 17 *NBS-LRR* genes were differentially expressed during the CCN infection, and 12 of them were down-regulated (Fig. 3; Table S5).

### Genes associated with metabolic and transporter progress were strongly affected by CCN infection

In this study, we found that the expression profiles of many genes that function in metabolic pathways were significantly affected by the CCN. For example, we observed that the genes encoding glucan endo-1,3-beta-glucosidase, beta-glucosidase, chitinase and acidic endochitinase in the carbohydrate metabolic process were up-regulated at 1 dpi and 3 dpi, while beta-fructofuranosidase, endoglucanase 12, beta-fructofuranosidase and sucrose: sucrose 1-fructosyltransferase genes were down-regulated. In the fatty acid biosynthetic process, the 3-ketoacyl-CoA synthase 11 gene was up-regulated while the long chain acyl-CoA synthetase 9 gene was down-regulated. Genes involved in the lipid metabolic process were up-regulated at 3 dpi. The tyrosine/DOPA decarboxylase 2, methionine gamma-lyase, and aromatic-L-amino-acid decarboxylase genes which are involved in the amino acid metabolic process were greatly up-regulated at 1 and 3 dpi (Fig. 3; Table S6). In addition, transporter genes were also greatly enriched in both up- and down-regulated genes, but the vesicle-mediated transport genes were only enriched in the down-regulated group at 1 dpi and 3 dpi. For example, the gene encoding sugar transport protein 4 was up-regulated at 1 and 3 dpi, and the gene encoding sugar carrier protein A was up-regulated at 3 dpi and 8 dpi. Genes encoding amino-acid permease BAT1, probable peptide/nitrate transporter, and peptide transporter PTR5 were significantly up-regulated at 1 dpi and 3 dpi. Genes encoding peptide transporter PTR2, aquaporin NIP1-1, probable purine permease 5, probable potassium transporter, auxin transporter-like protein 2 and probable auxin efflux carrier component 4 were significantly up-regulated at 1 dpi, 3 dpi and 8 dpi (Fig. 3; Table S7).

### GO enrichment of the up-regulated genes at all three time points

**Figure 4:**
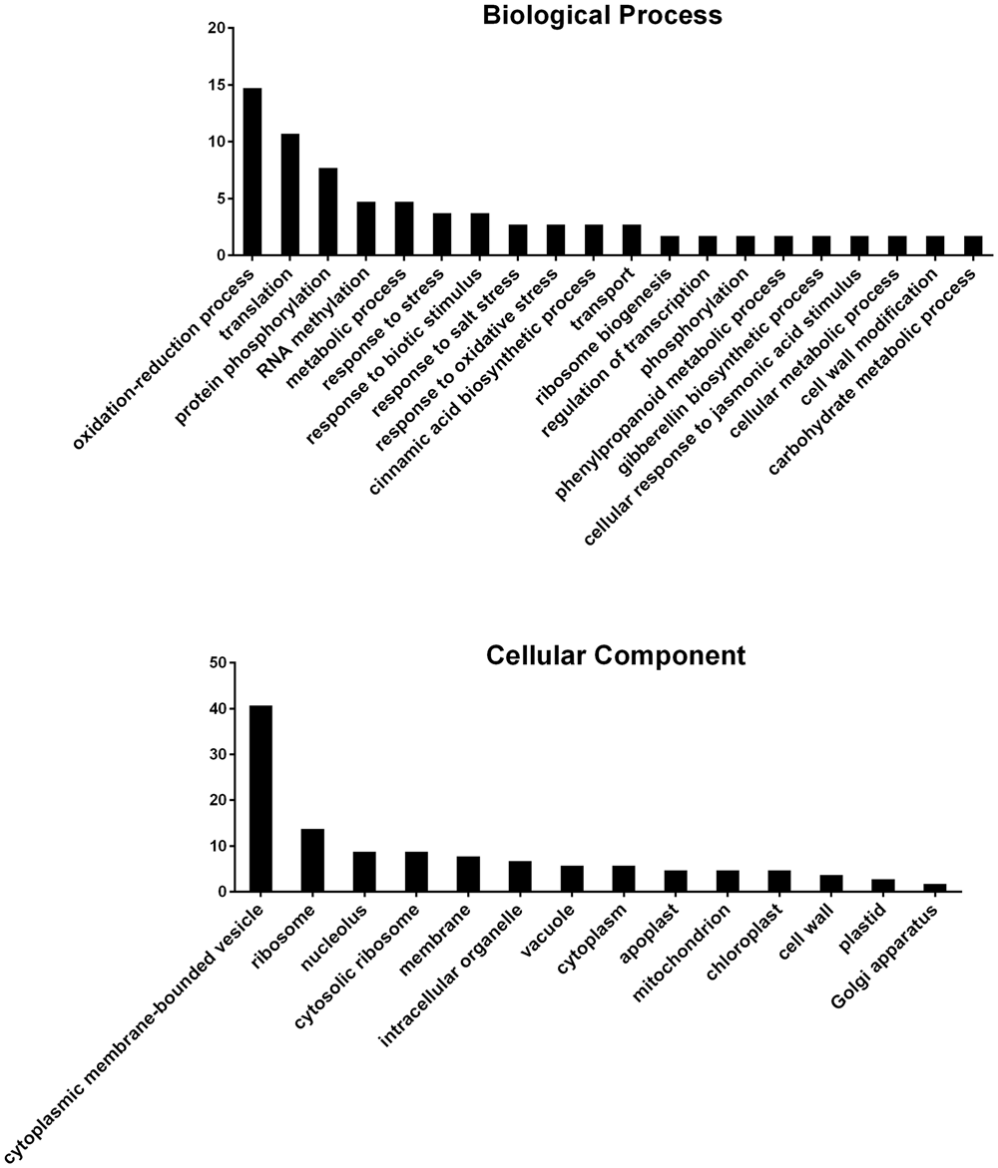
GO biological process and cellular component definition of up-regulated genes at all three time points (1 dpi, 3 dpi and 8 dpi). Y-axis indicates the numbers of genes.

There are some genes that were up-regulated at all three time points: 1 dpi, 3 dpi and 8 dpi. In total, 240 up-regulated transcripts were obtained. GO enrichment showed that a large number of the oxidation-reduction process, translation, protein phosphorylation, response to biotic stimulus, metabolic process and cell wall modification genes were found in the up-regulated group (Fig. 4a). Cellular component enrichment showed that the largest numbers of genes products were found in the cytoplasmic membrane-bounded vesicle (Fig. 4b). The first set of candidates related to wheat susceptibility to CCN were selected from 3 GO terms: protein phosphorylation, oxidation-reduction process, metabolic process (Table 4). The second set of candidates was selected among the strongest up-regulated genes including those for: vegetative cell wall protein, elongation factor 1-alpha, ent-kaurenoic acid oxidase 1, and the cytoskeleton related genes: actin, tubulin alpha chain, and tubulin beta chain (Table 4).

**Table 4:** Selected genes that were continuously up-regulated in the infected Wen 19 compared to the non-infected Wen 19 at all the time points (1 dpi, 3d pi and 8 dpi). FC: fold change (CCN infection *vs* non-infected samples).

GenBank-ID	Annotation	Log_2_FC 1 dpi	Log_2_FC 3 dpi	Log_2_FC 8 dpi
MH908122	Wall-associated receptor kinase 5	1.9181	4.234	1.756
MH908123	Wall-associated receptor kinase 3	1.5391	3.7122	2.0208
MH908124	Peroxidase 4	3.4673	4.3622	1.6753
MH908125	Peroxidase 23	3.853	2.3877	1.5927
MH908126	Probable flavin-containing monooxygenase 1	2.5031	10.894	8.3219
MH908127	2-oxoglutarate/Fe(II)-dependent dioxygenase	12.893	3.5903	1.9144
MH908128	Leucoanthocyanidin dioxygenase	11.7895	10.5855	2.3999
MH908129	Bifunctional monodehydroascorbate reductase	12.7432	11.7372	5.9681
MH908130	1-aminocyclopropane-1-carboxylate oxidase homologue 2	2.9182	10.5613	7.6675
MH908131	UDP-glucosyltransferase	11.7396	11.4498	1.4929
MH908132	Probable glutathione S-transferase GSTU6	1.836	3.0525	1.4407
MH908133	glutathione S-transferase 23	2.977	4.7771	2.3581
MH908134	Probable glutathione S-transferase GSTF2	2.4487	5.059	2.1934
MH908135	Elongation factor 1-alpha	11.1322	12.1807	11.0826
MH908136	Ent-kaurenoic acid oxidase 1	13.2378	11.1729	5.0316
MH908137	Actin	7.3949	11.5589	13.9831
MH908138	Tubulin alpha chain	10.3013	12.4728	11.6972
MH908139	Tubulin beta chain	9.1306	11.3262	10.623

## Discussion

In this study, we analysed the gene transcription levels in susceptible wheat Wen 19 during interaction with the CCN at 1 dpi, 3 dpi and 8 dpi which correspond to the stages of nematode penetration, feeding site establishment and maintenance, respectively^5,6^. We found 3649, 6029 and 1251 significant DEGs from evenly clean reads of 1 dpi, 3 dpi and 8 dpi samples, respectively (Table S1), suggesting that the Wen19 may have a strong reaction during the early infection stages (1 dpi and 3 dpi), but the nematode is able to inhibit the plant responses in the compatible interaction at 8dpi.

At 1 dpi, the J2s have penetrated the root^6^. Both the penetration and migration of J2 are dependent on the degradation and loosening of the host cell wall^16^. At 3 dpi, the syncytium is established^5,6^, possibly as a result of the modulation of the plant cell wall and hormones. For instance, cell wall modification and hydrolysis-related genes were induced, and auxin concentration, transportation and distribution were altered^9,22^. In this study, the gene set of plant-type cell wall was enriched at 1 dpi (Fig. 2a). Genes encoding xyloglucan endotransglycosylase/hydrolase protein, expansins, endoglucanase 2 and endoglucanase 4 were up-regulated by the CCN infection (Table 2), which was consistent with previous reports that the genes encoding expansins, endoglucanases and pectate lyases were up-regulated by nematode infection^9^. The gene set of the auxin-mediated signalling pathway was up-regulated at 3 dpi and 8 dpi (Fig. 2b,c). Genes involved in auxin biosynthesis such as those encoding flavin-containing monooxygenase YUCCA9, probable indole-3-acetic acid-amido synthetase, aromatic-L-amino-acid decarboxylase^25^ and auxin response, as well as induced proteins, were up-regulated by the CCN infection at 1 dpi and 3 dpi (Table 3), which was consistent with the previous report that auxin was a key element for syncytium formation^22^. An auxin influx carrier gene was significantly up-regulated, but a probable auxin efflux carrier component 1c gene was down-regulated (Table 3). A similar result that auxin transporter PIN was important for CCN infection has been reported^26^.

Metabolic activity and nutrient transport play an important role in CCN infection^27,28^. GO terms of biological process enrichment showed that the genes involved in the metabolic process, protein phosphorylation, and transport progression were greatly enriched in both up- and down-regulated genes during the CCN infection. It was reported that metabolic and transport progress was very active in the syncytia^27,28^. We found that in the carbohydrate metabolic process, the genes encoding glucan endo-1,3-beta-glucosidase and beta-glucosidase were up-regulated (Fig. 3; Table S6), which suggested that polysaccharide catabolism was induced. Simultaneously, the genes encoding the sugar transport protein 4 and sugar carrier protein A were up-regulated (Fig. 3; Table S6), indicating a profound role of sugar transportation in the CCN infection. This result is consistent with previous reports that the content of soluble sugars in the stems of tomato has increased after RKN infestation^29^. Sugar transporters were specifically expressed and active in the syncytia in the previous studies^30^. In this study, we found that amino acid, peptide metabolic and transporter genes were greatly induced by the CCN (Table S7), which suggests that amino acid metabolism plays a crucial role for nematode infection. Previous metabolic profiling revealed that amino acids and phosphorylated metabolites were increased in the syncytia^28^. In addition, we found that aquaporin NIP1-1 genes (Table S7), which are associated with stress tolerance^31^, were significantly induced by CCN.

Nematode invasion may have induced basal host resistance^32,33^. We found that several genes associated with wounding response were up-regulated at 1 dpi and 3 dpi (Fig. 2a,b), which may be a result of the penetration and migration of CCN. It has been reported that damage-induced defence is controlled by the JA signalling pathway^34^. In our dataset, the genes that respond to jasmonic acid stimulus were enriched in up-regulated GO terms at 1 dpi (Fig. 2a). In addition, the probable linoleate 9S-lipoxygenase 4 gene was intensely induced at 1 dpi but not in the later infection stages. The allene oxide cyclase 3 gene was induced only at 3 dpi (Table 3), which may indicate that the host induces JA response at the early stage of CCN infection. In the interactions of *Arabidopsis thaliana* and *H. schachtii*, JA-triggered early defence has been reported^35^. In tomato, the JA signalling pathway is required during compatible interaction with RKN^36^. In addition, we found that the respiratory burst oxidase genes were induced by CCN infection at 3 dpi (Table S3). Early host defence, including ROS induction, was reported during the compatible interactions of *Musa acuminata* with RKN^15^. Alternatively, the negative regulation of the defence progress was up-regulated (Fig. 2a), and the response to oxidative stress genes was down-regulated at 1 dpi (Fig. 2d), suggesting that the resistance reaction was under control. The antioxidant enzymes peroxidase, ascorbate and anthocyanidin reductase were induced by CCN. Pioneering research illustrated that the overexpression of anthocyanidin reductase increased ROS scavenging and enhanced tobacco tolerance^37^. It was reported that the effector MjTTL5 from the RKN can interact with ferredoxin to dramatically activate ROS scavenging, resulting in defence suppression^38^. In this study, the genes encoding UDP-glycosyltransferase and glutathione S-transferases (GSTs) were highly expressed after CCN infection, suggesting that they may play an important role in the wheat-CCN interaction. GSTs play conserved roles in the detoxification of toxic compounds from pathogen attack and oxidative stress^39^. The RKN and pine wood nematode can secrete GSTs into their hosts to counteract plant resistance^40,41^. In compatible soybean, GST was induced in the syncytia and whole roots by the soybean cyst nematode^42^. In this study, 22 of 24 GST genes were significantly up-regulated after nematode infection at 3 dpi. We also found that the UDP-glycosyltransferase genes were significantly up-regulated after CCN infection at 1 dpi and 3 dpi. The overexpression of the UDP-glucosyltransferase from *A. thaliana* can improve plant tolerance^43^. The UDP-glucosyltransferase from *Brachypodium distachyon* can detoxify deoxynivalenol from *Fusarium graminearum*^44^. Antioxidant enzymes, such as GSTs and UDP-glycosyltransferase, may be crucial in scavenging ROS and detoxifying toxins in wheat after nematode attack, as well as keeping the plant defence under control. We also found that most *NBS-LRR* genes were down-regulated, illustrating the critical role of resistance suppression in successful CCN infection. The suppression of tomato defense responce genes upon potato cyst nematode infection indicates a key regulatory role of miRNAs^45^. Successful nematode parasitism appears to involve recognition by the host, the induction of early resistance reactions, the elimination of ROS and control of resistance^46^. Cloning the enzyme-encoding genes and verification of their functions will further improve our understanding of the CCN infection and could help to the design of effective management strategies to reduce CCN damage.

In conclusion, from an analysis of compatible wheat-cereal cyst nematode interaction analyses, a large number of genes and pathways associated with CCN infection were revealed. In this study, we found that the metabolic and transporter processes were regulated by CCN. This process may facilitate the development of the nematodes. Plant cell wall hydrolysis, modifying proteins and auxin biosynthesis, signalling and transporter genes were regulated by the nematode. This may facilitate the penetration, migration and syncytium establishment. It seems that CCN infection can induce host defense reactions but can also hijack the antioxidant and detoxification processes of the plant to eliminate ROS and toxic components and suppress the *NBS-LRR* resistance gene expression.

## Materials and Methods

### Plant materials and CCN treatments

A compatible wheat variety *Triticum aestivum* L. cv. Wen 19 was used for transcriptomic profiling in this study. The seeds were germinated in Petri dishes on wet gauze at 25 °C in the dark for 2 days. Seedlings were planted in 50 mL tubes filled with sterilized sands and grown at 16 °C under a 16 h light/8 h dark photoperiod for 7 days^6^. Cereal cyst nematodes were propagated in the greenhouse under non-sterile conditions as previously described^47^. Fully developed cysts were incubated in 3 mM ZnCl_2_, and the J2 larvae were harvested and counted with a microscope. Each seedling was inoculated with 500 J2 larvae and the negative control was not inoculated. At 24 hours past inoculation, the roots were washed three times with sterilized H_2_O to remove CCN adhering to the roots, transplanted into pots containing 500 mL of sterilized sands and grown as described by Kong *et al*. and Xu *et al*.^6,48^.

### RNA extraction and sequencing

Root materials were sampled at 1, 3 and 8 days past inoculation, and immediately frozen in liquid nitrogen. Total RNA was extracted using the TRIzol reagent according to the manufacturer’s instructions and treated with DNase (Invitrogen, USA). The concentration and quality of the RNA was determined using a NanoDrop-2000 (ThermoFisher Scientific Inc., USA). RNA extracted at 1 dpi, 3 dpi and 8 dpi were quantified to an equal concentration by adding RNase-free water and subsequently sequenced (paired-end, 2 × 100 bp) using an Illumina HiSeq™ 2000 (Illumina Inc., USA).

### Bioinformatic analysis

The data analysis was performed as previously described^6^. During the reads trimming, low quality reads (Phred score <Q30), reads containing more than two ambiguous bases (‘N’) and reads shorter than 50 nucleotides were filtered out. The CCN transcripts were removed as previously described^6^. The clean reads from each sample were *de novo* assembled together using the Trinity program package^49^. Gene annotation was performed *via* BLASTx alignments to protein databases (Nr, Swiss-Prot, KEGG and COG) and BLASTn alignments to the NCBI nucleotide databases with a cut-off E-value of 1e^−5^. We performed the GO annotations of the transcripts by Blast2GO program^50^. The expressions of unigenes were calculated by Fragments Per kb per Million Fragments (FPKM). The false discovery rate (FDR) was used to determine the *P* value threshold. A gene was judged to be a DEG if the *P* value (FDR) ≤0.001 and the fold change (FC) between CCN infection and control samples at each time point ≥2^51^.

## Supplementary information

Dataset 1

## Data Availability

Sequences of differentially expressed transcripts were submitted to GenBank and GenBank accession numbers are provided in Tables 2, 3 and 4.
