# Supplementary material for: Transcriptional profiling of wheat (Triticum aestivum L.) during a compatible interaction with the cereal cyst nematode Heterodera avenae
Source: Sci Rep. 2019 Feb 18;9:2184. doi: 10.1038/s41598-018-37824-9 (PMC6379437; doi:10.1038/s41598-018-37824-9)
Supplement: Supplementary file 1 — Dataset 1 [file 41598_2018_37824_MOESM1_ESM.pdf]

Transcriptional profiling of wheat (*Triticum aestivum* L.) during a compatible interaction with the cereal cyst nematode *Heterodera avenae*

Fen Qiao[1#], Ling-An Kong[1#], Huan Peng[1], Wen-Kun Huang[1], Du-Qing Wu[1], Shi-Ming Liu[1], Jihong Liu Clarke[2], De-Wen Qiu[1], De-Liang Peng[1*].

Supplementary Tables

Table S1: Gene expression analyses of Wen19 in response to CCN by RNA-Seq. Differentially expressed genes (DEGs) between non-infected and infected Wen19 at 1dpi, 3dpi and 8dpi were listed

CCN response and control	No. of clean Reads	No. of DEGs	No. of DEGs up-regulated	No. of DEGs down-regulated
1 dpi	214,092,190	3649	2513	1136
3 dpi	215,995,184	6029	4467	1562
8 dpi	215,455,900	1251	945	306
total	645,543,274	10929	7925	3004

Table S2: Genes greatly up/down-regulated of infected Wen19 compared to the non-infected at 1dpi, 3dpi and 8dpi. FC: fold change (CCN infected vs non-infected samples); --: not found.

Wheat-ID	Swissprot-annotation	Log_2FC	Pathway
1dpi			
Traes_5DL_7CC241DA0.1	Aromatic-L-amino-acid decarboxylase	13.1333	tyrosine decarboxylase
Traes_4AL_2B70B04B6.2	Beta-sesquiphellandrene synthase	12.3512	beta-farnesene synthase
Traes_1BL_E5E8D9BC9.1	Formin-like protein 8	12.1639	solute carrier family 39
Traes_3B_8036ADEB6.1	Probable linoleate 9S-lipoxygenase 4	11.2279	response to JA stimulus
Traes_4AS_19FA06316.1	Nodulation-signaling pathway 2 protein	11.4278	regulation of transcription
Traes_4DS_CA2D872D6.1	UDP-glycosyltransferase 91B1	10.6547	metabolic process
Traes_5DL_91AE6CA271.4	Dehydration-responsive element-binding protein 1H	-10.5458	regulation of transcription
Traes_1AL_65A99675F.1	Senescence-induced receptor-like serine/threonine-protein kinase	-10.2126	--
3dpi			
Traes_2DS_1063CD755.1	Probable glutathione S-transferase GSTF1	12.7813	glutathione S-transferase
Traes_4BL_98B0C2E78.1	Bidirectional sugar transporter SWEET12	12.6425	solute carrier family 50
Traes_2DS_D2EF60FE1.3	Ethylene-responsive transcription factor RAP2-2	11.675	--
Traes_6DL_6EC763C47.1	Peptide transporter PTR2	10.5579	transport
Traes_5DL_C65153D64.1	indole-2-monooxygenase	10.7533	oxidation-reduction process
Traes_3B_82E1F5484.1	Ethylene-responsive transcription factor TINY	-11.2707	EREBP-like factor
Traes_6DS_D1697BDF3.1	Transcription factor HY5	-9.4566	ABA responsive element binding factor
Traes_1AL_CC827E5C6.1	Phytochrome-associated serine/threonine-protein phosphatase	-8.2095	protein dephosphorylation;
8dpi			
Traes_1AL_F18C11AB1.1	Cytosolic sulfotransferase 12	12.6623	desulfoglucosinolate sulfotransferase A/B/C
Traes_2BL_9AB7B773F.1	Vegetative cell wall protein gp1	12.1175	phospholipase D
Traes_7DL_8650816CA.2	Proline-rich receptor-like protein kinase PERK10	11.5405	ataxia telangiectasia mutated family protein
Traes_1AL_258E7F2A3.1	ethylene responsive transcription factor	10.2378	regulation of transcription
Traes_4DL_7A2335529.2	uncharacterized protein	-10.5519	--
Traes_1DL_E2E9F90E4.2	Beta-galactosidase 7	-3.0598	carbohydrate metabolic process
Traes_4BL_10B3124F1.1	Elongation factor 1-alpha C	-4.3825	translational elongation process

Table S3: Expression levels of genes for respiratory burst oxidase and antioxidant enzyme genes in the infected Wen19 compared to the non-infected at 1dpi, 3 dpi and 8 dpi by RNA-seq. FC: fold change (CCN infected vs non-infected samples); --: not differentially expressed.

Wheat-ID	log_2FC 1dpi	log_2FC 3dpi	log_2FC 8dpi	Swissprot-annotation
respiratory burst oxidase				
Traes_1DL_0C3B0EA5C.1	1.4745	1.3092	-1.3179	Respiratory burst oxidase homolog protein [*Arabidopsis thaliana*]
Traes_5BL_CF34FCA90.3	--	1.5787	--	Respiratory burst oxidase homolog protein C [*Arabidopsis thaliana*]
Traes_1AL_D25D2A8D7.2	2.9663	2.6897	2.6283	Respiratory burst oxidase homolog protein D [*Arabidopsis thaliana*]
Traes_1DL_5B3CEB047.1	2.1291	11.254	3.3257	Respiratory burst oxidase homolog protein B [*Arabidopsis thaliana*]
Traes_5DS_2809B6F77.4	--	2.7678	--	Respiratory burst oxidase homolog protein B [*Arabidopsis thaliana*]
Traes_5DS_2809B6F77.4	--	2.5263	--	Respiratory burst oxidase homolog protein B [*Arabidopsis thaliana*]
Traes_5BL_CF34FCA90.1	--	1.5687	--	Respiratory burst oxidase homolog protein D [*Arabidopsis thaliana*]
Traes_5DS_2809B6F77.3	--	3.1171	--	Respiratory burst oxidase homolog protein B [*Arabidopsis thaliana*]
antioxidant enzymes				
Traes_5BS_BA048F803.1	2.4348	10.4995	--	Peroxidase [*Triticum aestivum*]
Traes_2BL_A25152F2A.1	1.1543	2.0879	--	Peroxidase 70 OS=Zea mays GN=PER70 PE=1 SV=3
Traes_2BL_A25152F2A.1	1.4658	4.2906	1.8623	Peroxidase 52 [*Arabidopsis thaliana*]
Traes_1DS_103FB69FD.1	2.6368	4.9846	2.5444	Peroxidase 5 [*Vitis vinifer*a]
Traes_4DS_0B056789B.2	3.6234	3.3004	1.7683	Peroxidase 4 [*Vitis vinifera*]
Traes_2BL_A25152F2A.2	1.1907	4.852	1.6419	Peroxidase 2 [*Oryza sativa* subsp. Japonica]
Traes_3AL_EB37D49001.1	3.2131	2.2517	1.493	Peroxidase 15 [*Ipomoea batatas*]
Traes_2DL_FFB73A9AD.1	4.7429	2.333	--	Cationic peroxidase SPC4 [*Sorghum bicolor*]
Traes_6DS_3522B8EF6.1	1.0087	1.5313	--	Catalase isozyme 2 [*Hordeum vulgare*]
Traes_2DL_F2EBB4B14.1	2.8716	1.6859	-1.3189	Peroxidase 56 [*Arabidopsis thaliana*]
Traes_1BS_BA046E212.1	-2.5092	2.5995	1.9563	Peroxidase 54 [*Arabidopsis thaliana*]
Traes_4AL_89CCB25EA.1	-3.0777	-1.5909	1.2907	Peroxidase 2 [*Zea mays*]
Traes_2DL_1C4C67BC8.1	1.9136	1.6992	--	Peroxidase 12 [*Arabidopsis thaliana*]
Traes_7DL_6EFFDA6D1.1	1.3741	2.4277	--	--
Traes_5BL_04D10C4E7.1	2.3112	11.0018	--	L-ascorbate oxidase [*Cucurbita pepo* var. melopepo]
Traes_2DL_A55995202.2	8.0482	11.2121	--	Anthocyanidin reductase [*Arabidopsis thaliana*]
Traes_7AL_4842B84BF.2	--	2.1176	1.0148	Anthocyanidin reductase [*Arabidopsis thaliana*]

Table S4: Expression levels of UDP-glucosyltransferase and glutathione S-transferases (GST) genes in the infected Wen19 compared to the non-infected at 1dpi, 3 dpi and 8 dpi by RNA-seq. FC: fold change (CCN infected vs non-infected samples); --: not differentially expressed.

Wheat-ID	log_2FC 1dpi	log_2FC 3dpi	log_2FC 8dpi	Swissprot-annotation
UDP-glucosyltransferase				
Traes_5BL_78AFC51C81.2	11.7396	11.4498	1.4929	UDP-glucosyltransferase [*Arabidopsis thaliana*]
Traes_2DS_DBE32D98B.1	6.1412	7.5069	1.4879	UDP-glucosyltransferase [*Arabidopsis thaliana*]
Traes_6DS_EC682031E.1	2.2077	4.1919	--	UDP-glycosyltransferase 85A5 [*Arabidopsis thaliana*]
Traes_5DS_19AE064C1.1	2.121	3.5311	--	UDP-glycosyltransferase 74E1 [*Arabidopsis thaliana*]
Traes_5AL_BCA187DFF.1	1.5686	1.3604	--	UDP-glycosyltransferase 74E2 [*Arabidopsis thaliana*]
Traes_4DS_CA2D872D6.1	1.3333	2.8595	--	UDP-glycosyltransferase 91C1 [*Arabidopsis thaliana*]
Traes_6DL_4AC316C93.1	1.3332	3.0433	--	UDP-glycosyltransferase 85A1 [*Arabidopsis thaliana*]
Traes_3B_A2CE643F8.1	1.2194	1.6582	--	UDP-glycosyltransferase 73C5 [*Arabidopsis thaliana*]
Traes_2BS_48735B0CB.1	1.2076	4.3534	-1.337	UDP-glycosyltransferase 76C2 [*Arabidopsis thaliana*]
Traes_5BL_DD0352E4C.1	1.182	1.4972	--	UDP-glycosyltransferase 90A1 [*Arabidopsis thaliana*]
Traes_5BL_3C4FA0AE5.2	1.0513	1.5491	--	UDP-glycosyltransferase 75D1 [*Arabidopsis thaliana*]
Traes_5DL_F1C1DEBBF.1	--	3.8594	1.3999	UDP-glycosyltransferase 73B4 [*Arabidopsis thaliana*]
Traes_2BL_C6981594D.1	--	1.2747	--	UDP-glycosyltransferase 72B1 [*Arabidopsis thaliana*]
Traes_1AS_B486CB8E2.1	--	10.3536	1.985	UDP-glycosyltransferase 85A3 [*Arabidopsis thaliana*]
Traes_6DL_E2E8047EE.1	--	-1.1903	--	UDP-glycosyltransferase 85A2 [*Arabidopsis thaliana*]
glutathione S-transferases (GST)				
Traes_1AL_CA2AFD745.1	4.1148	2.8059	--	Probable GSTU6 [*Oryza sativa* subsp. Japonica]
Traes_1AL_C40703A29.1	3.0701	1.4673	2.0779	Probable GSTU6 [*Oryza sativa* subsp. Japonica]
Traes_5DL_58B23C5A4.3	2.977	4.7771	2.3581	GST 2 *[Triticum aestivum*]
Traes_4AL_347F5BB64.1	2.4487	5.059	2.1934	Probable GSTF2 [*Oryza sativa* subsp. Japonica]
Traes_5BL_2D1D16169.2	1.977	2.5496	--	Probable GSTF1[*Oryza sativa* subsp. Japonica]
Traes_7AS_B97EB9A75.1	1.9593	2.8619	--	Probable GST *[Nicotiana tabacum*]
Traes_1DS_B686BC1E0.1	1.836	3.0525	2.2012	Probable GSTU6 *[Oryza sativa* subsp. Japonica]
Traes_3B_D47C46EC2.1	1.7179	2.0955	--	GST 3 [*Zea mays*]
Traes_2BS_26A877813.1	1.6552	2.6898	2.0779	Probable GSTU6 [*Oryza sativa* subsp. Japonica]
Traes_5BS_D1BA3D7D3.1	1.6117	1.7701	--	GST 4 [*Zea mays*]
Traes_2BL_54C69C35D.1	1.3181	2.553	1.2434	Probable GST [*Nicotiana tabacum*]
Traes_5BL_E956B149A.1	1.2831	3.1047	--	GST 23 [*Zea mays*]
Traes_3B_1B7EAAB22.1	--	4.7483	1.3735	GST 23 [*Zea mays*]
Traes_2DS_1063CD755.1	--	12.7813	2.0166	Probable GSTF1 [*Oryza sativa* subsp. Japonica]
Traes_1AL_2103C5913.1	--	1.4584	--	GST U17 [*Arabidopsis thaliana*]
Traes_5BL_6E5FBD11D.5	--	1.2816	--	GST [*Triticum aestivum*]
Traes_2DL_311457A3D.2	--	1.7162	--	GST U19 [*Arabidopsis thaliana*]
Traes_4AL_A06C88922.2	--	5.4338	1.2895	Probable GSTU6 [*Oryza sativa* subsp. Japonica]
Traes_3DL_B781DEF97.1	--	1.3345	--	GST 1 [*Zea mays*]
Traes_2AL_24740AC23.1	--	1.6706	--	GST 3 [*Glycine max*]
Traes_5DL_D7DD6CA07.1	--	-3.4019	--	GST [*Arabidopsis thaliana*]
Traes_3B_F1C4DE560.1	--	2.6073	1.9322	Probable GST [*Glycine max*]
Traes_2DL_87838170E.1	--	3.0958	1.0985	Probable GST [*Nicotiana tabacum*]
Traes_3DL_8D15D0EF8.1	-1.5888	-1.3358	--	GST U8 [*Arabidopsis thaliana*]

Table S5: Expression levels of NBS-LRR genes in the infected Wen19 compared to the non-infected at 1dpi, 3 dpi and 8 dpi by RNA-seq. FC: fold change (CCN infected vs non-infected samples); --: not differentially expressed.

Wheat-ID	log_2FC1 dpi	log_2FC 3dpi	log_2FC 8dpi	Nr-annotation
Traes_2BS_57256616B.4	--	3.275	2.0782	NBS-LRR R protein [*Hordeum vulgare*]
Traes_6DL_165369544.1	-1.9301	1.8703	--	NBS-LRR R protein [*Setaria italica*]
Traes_7BS_9716221D5.1	--	1.6897	--	NBS-LRR type R protein [*Cynodon magennisii*]
Traes_5DL_51EF17022.3	1.6557	1.5198	1.2479	NBS-LRR R protein family-3 [*Oryza brachyantha*]
Traes_2DL_FFC3E4F86.1	--	1.4671	1.537	NBS-LRR R protein [*Hordeum vulgare*]
Traes_7BL_1C6415BB2.3	--	-1.216	-1.5088	NBS-LRR-like protein [*Hordeum vulgare* subsp. vulgare]
Traes_2BS_26B28D804.1	-1.5147	-1.8957	1.815	NBS-LRR R protein [*Hordeum vulgare* subsp. spontaneum]
Traes_1AS_E6F253266.3	--	-1.9828	-1.1443	NBS-LRR R protein [*Hordeum vulgare*]
Traes_1AS_E6F253266.2	--	-2.0471	-1.1026	NBS-LRR R protein [*Hordeum vulgare*]
Traes_7AS_E43D64A80.6	--	-2.2172	--	NBS-LRR resistance-like protein *[Hordeum vulgare*]
Traes_6BS_27BE3E745.16	--	-2.4572	--	NBS-LRR R protein [*Hordeum vulgare*]
Traes_2DL_ADACB74CD.2	--	-2.4793	--	NBS-LRR R protein [*Hordeum vulgare*]
Traes_6BS_27BE3E745.4	--	-2.7028	1.2155	NBS-LRR R protein [*Hordeum vulgare*]
Traes_4AL_2DE434D73.4	-1.1526	-3.0647	1.2479	NBS-LRR-like protein [*Hordeum vulgare* subsp. vulgare]
Traes_7DS_A7A26AAA4.1	1.0087	-3.4184	--	NBS-LRR R protein [*Hordeum vulgare*]
Traes_2BS_E7B6E117A.1	--	-4.5382	--	NBS-LRR type protein [*Oryza sativa* Indica Group]
Traes_1AS_860F1C549.1	1.0701	-9.9275	--	NBS-kinase-like protein [*Secale strictum* subsp. kuprijanovii]

Table S6: Expression levels of some genes in metabolic pathways in the infected Wen19 compared to the non-infected at 1dpi, 3 dpi and 8 dpi by RNA-seq. FC: fold change (CCN infection vs control samples); --: not differentially expressed.

Wheat-ID	log_2FC 1dpi	log_2FC 3dpi	log_2FC 8dpi	Swissprot-annotation
Traes_3DS_3D22DDF16.1	-8.4355	-1.9308	--	Endoglucanase 12 [*Oryza sativa* subsp. Japonica]
Traes_6DS_204661A07.1	-1.2577	-1.4007	--	Beta-fructofuranosidase 1 [*Zea mays*]
Traes_6DL_61AA02A3D.2	-1.5493	-1.5592	--	Beta-D-xylosidase 3 [*Arabidopsis thaliana*]
Traes_3AL_DCFA76D6D.1	1.7024	11.0614	3.3997	class III chitinase [*Bambusa oldhamii*]
Traes_1BL_081C896A0.1	2.209	2.2838	--	chitinase 2 precursor [*Zea mays*]
Traes_7AL_FAE816A85.1	2.4677	2.8637	1.3259	chitinase 2-like [*Brachypodium distachyon*]
Traes_1BL_081C896A0.1	1.8406	2.4527	--	Chitinase [*Oryza sativa* Japonica Group]
Traes_4AS_5535CF08E.1	1.392	1.3876	--	Beta-glucosidase 6 [*Oryza sativa* subsp. Japonica]
Traes_2BS_2EDCB210E.3	10.8887	8.7672	-8.1174	Beta-glucosidase 26 [*Oryza sativa* subsp. Japonica]
Traes_2AL_D3D671F67.3	1.2277	1.1375	--	Beta-glucosidase 12 [*Oryza sativa* subsp. Japonica]
Traes_3B_EDFCBD301.1	2.1666	13.2378	--	beta-1,3-glucanase precursor [*Triticum aestivum*]
Traes_3B_EDFCBD301.1	1.4027	12.5468	--	beta-1,3-glucanase 2a [*Zea mays*]
Traes_2DS_721623E22.1	0.0258	2.6695	1.636	Glucan endo-1,3-beta-glucosidase 7 [*Arabidopsis thaliana*]
Traes_2BS_9C52B176E.4	0.4033	2.3227	1.0322	Glucan endo-1,3-beta-glucosidase 3 [*Arabidopsis thaliana*]
Traes_2BS_9C52B176E.1	0.8826	6.0705	2.3003	Glucan endo-1,3-beta-glucosidase 13 [*Arabidopsis thaliana*]
Traes_4AL_9D7ECD3BA.1	-1.1531	-1.1671	--	Long chain acyl-CoA synthetase 9 [*Arabidopsis thaliana*]
Traes_5DS_DAF26AAD9.1	1.5677	1.5555	--	3-ketoacyl-CoA synthase 11 [*Arabidopsis thaliana*]
Traes_3AS_77CBFA352.1	1.0701	2.0242	--	GDSL esterase/lipase [*Arabidopsis thaliana*]
Traes_2BL_424BDA37F.2	13.1333	2.6898	--	Aromatic-L-amino-acid decarboxylase [*Catharanthus roseus*]
Traes_7AL_0F8FA764F.1	6.0844	4.9628	2.3992	Aromatic-L-amino-acid decarboxylase [*Catharanthus roseus*]
Traes_3B_32AE0BA64.1	3.6609	2.2747	--	Tyrosine/DOPA decarboxylase 2 [*Papaver somniferum*]

Table S7: Expression levels of genes in transporter pathways in the infected Wen19 compared to the non-infected at 1dpi, 3 dpi and 8 dpi by RNA-seq. FC: fold change (CCN infection vs control samples); --: not differentially expressed.

Wheat-ID	log_2FC 1dpi	log_2FC 3dpi	log_2FC 8dpi	Swissprot-annotation
Traes_2BS_7DF366FE1.4	2.5985	1.8073	--	Sugar transport protein 4 [*Arabidopsis thaliana*]
Traes_5AL_FC472994F.1	--	2.2257	1.4766	Sugar carrier protein A [*Ricinus communis*]
Traes_5BL_3BD2C8DF3.2	--	-1.2851	-1.0795	Non-specific lipid-transfer protein-like [*Arabidopsis thaliana*]
Traes_4BL_82F5E9A41.1	--	2.4978	1.3964	Non-specific lipid-transfer protein 4.3 [*Hordeum vulgare*]
Traes_4AL_58F9FBC2E.1	--	2.2923	1.537	Non-specific lipid-transfer protein 4 [*Oryza sativa* subsp. Japonica]
Traes_2AS_5884229E4.1	-1.3622	--	--	Non-specific lipid transfer protein-like 1 [*Oryza sativa* subsp. Japonica]
Traes_5BL_53FCAF30F.1	2.3332	2.6282	--	Probable peptide/nitrate transporter [*Arabidopsis thaliana*]
Traes_7DL_9E0C74527.2	1.2077	3.031	--	Probable peptide/nitrate transporter [*Arabidopsis thaliana*]
Traes_7AS_DE627AE1C.1	--	2.5143	1.1254	Probable nitrite transporter [*Arabidopsis thaliana*]
Traes_2BL_B4193BAEF.2	1.9047	1.1788	-1.6389	Peptide transporter PTR5 [*Arabidopsis thaliana*]
Traes_6DL_CBDD44DA9.1	2.9182	3.8598	2.2478	Peptide transporter PTR2 [*Arabidopsis thaliana*]
Traes_6DL_6EC763C47.1	2.3919	10.5579	8.4553	Peptide transporter PTR2 [*Arabidopsis thaliana*]
Traes_2BL_54FA866FD.1	--	2.29	--	Amino acid permease 6 [*Arabidopsis thaliana*]
Traes_3AS_5379B266E.1	--	1.3171	--	Amino acid permease 3 [*Arabidopsis thaliana*]
Traes_1DL_F70395059.1	--	2.015	1.6046	auxin influx carrier (AUX1 LAX family) [*Oryza sativa* subsp. japonica]
Traes_7DS_A92314B8C.3	3.7952	10.0454	10.3063	Aquaporin NIP1-1 [*Oryza sativa* subsp. Japonica]
Traes_4DL_AB2084483.2	10.3512	2.1048	2.6638	Pleiotropic drug resistance protein [*Nicotiana plumbaginifolia*]
Traes_7DL_AEAE90568.1	3.3287	1.7262	--	pleiotropic drug resistance protein 2 [*Oryza sativa* subsp. Japonica]
